# CT features of lung agenesis – a case series (6 cases)

**DOI:** 10.1186/s12880-018-0281-5

**Published:** 2018-10-30

**Authors:** Jamshid Sadiqi, Hidayatullah Hamidi

**Affiliations:** Department of Radiology, French Medical Institute for Mothers & Children (FMIC), behind Kabul Medical University, Jamal mina, P.O. Box: 472, Kabul, Afghanistan

**Keywords:** Pulmonary agenesis- pulmonary aplasia- pulmonary hypoplasia

## Abstract

**Back ground:**

Lung agenesis is a rare congenital anomaly. The main etiology of the disease is unknown whereas genetic, iatrogenic and viral factors as well as vitamin A deficiency during early pregnancy may result in developmental failure of primitive lung bud causing unilateral pulmonary agenesis. Affected patients usually present with variable respiratory symptoms and recurrent chest infection at any age. Plain film demonstrates opaque unilateral lung while chest CT scan can definitely diagnosis the disease. The anomaly has three types. Type I is pulmonary agenesis, type II is called pulmonary aplasia and type III is pulmonary hypoplasia.

**Cases’ presentation:**

Six patients with main complaint of dyspnea underwent contrast enhanced chest CT in radiology department of French Medical Institute for Mothers and children, Kabul and were diagnosed lung agenesis.

Three patients were categorized as type II pulmonary agenesis (aplasia). Two patients, three months old boy and a seven year- old girl demonstrated right lung aplasia. Another patient boy of eighteen years old presented with left lung aplasia.

Two boys of four and seven months of age were classified as type I pulmonary agenesis (agenesis).

A boy of one year old was diagnosed pulmonary agenesis type III, right lung hypoplasia.

**Conclusion:**

Six patients were diagnosed with pulmonary agenesis by Chest CT scan. The clinicians should consider possibility of congenital pulmonary agenesis in dyspneic patients with opaque unilateral hemithorax in plain film.

## Background

Pulmonary agenesis is an extremely rare congenital entity which can occur unilateral or bilaterally. Almost all cases are unilateral since bilateral agenesis in not compatible with life [1]. Unilateral pulmonary agenesis for the first time was discovered by Depozze in a female autopsy in 1673 [2]. In 50% of cases, lung agenesia accompanies other congenital anomalies like cardiovascular [3], central nervous system [4] gastrointestinal, genitourinary system, skeletal system, Down syndrome and Klippel Feil syndrome [5]. The clinical features vary from asymptomatic to variable respiratory complaints such as dyspnea and respiratory distress with history of recurrent chest infections. The symptoms may occur as early as in neonatal period or later on during childhood and even adult life [1–5]. Physical examination of patients shows asymmetrical chest wall movements with absent or decrease respiratory sounds in unilateral hemithorax [5]. Chest x-ray demonstrates white-out hemithorax whereas further examinations like chest CT scan, bronchoscopy, bronchography and pulmonary angiography are needed for definitive diagnosis [6]. The treatment is often medical however surgical intervention may be required for some cases especially when other congenital anomalies coexist [7]. Here we present six patients from 1 month old to 18 years of age with different types of pulmonary agenesis.

## Case presentation

Six patients whom underwent contrast enhanced chest CT scan in radiology department of French Medical Institute for Mothers and children in Kabul were diagnosed lung agenesis during 2015 to 2018.

**Fig. 1:**
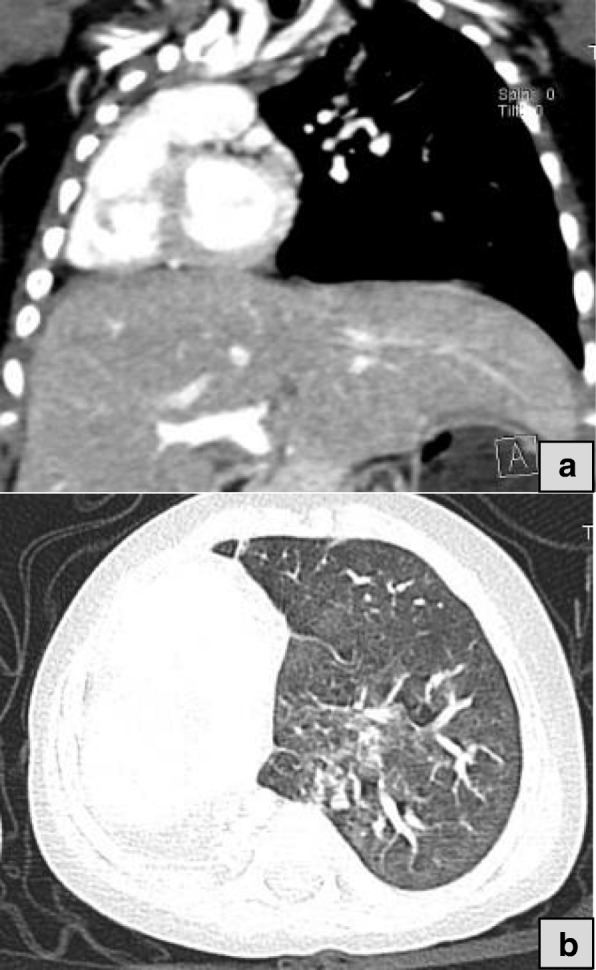
**a** Coronal CT image in mediastinal window shows complete absent of right lung, bronchus and vessels with complete heart and mediastinal shift into the right hemithorax. Fig. 1:**b** CT axial image in lung window demonstrates absence of right lung parenchyma with its vasculature and location of heart and great vessels in the right side. (Four months’ boy)

**Fig. 2:**
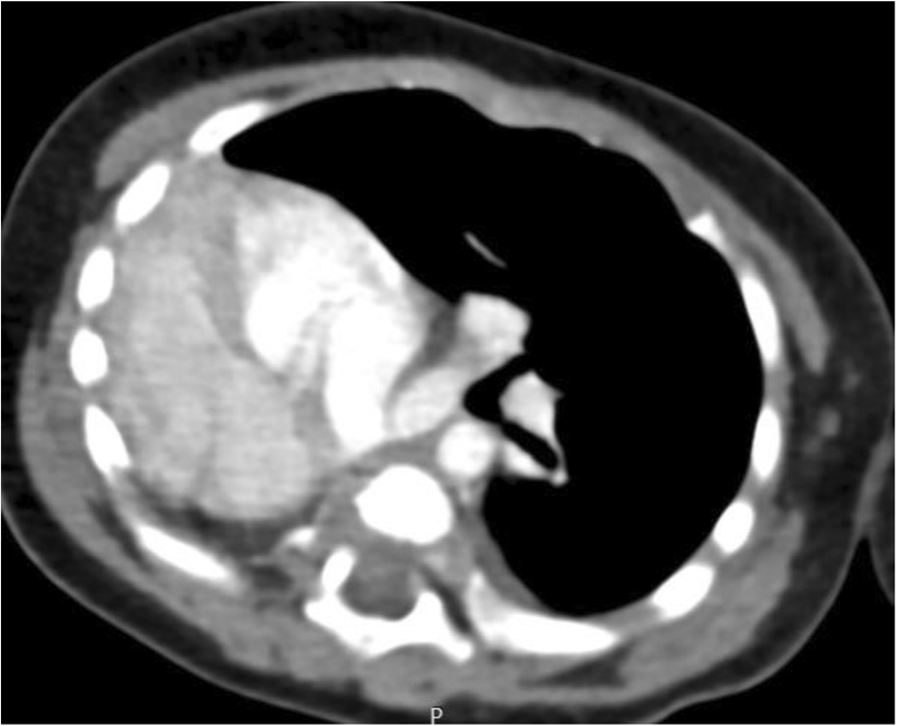
Mediastinal window CT image in axial section shows complete absent of right lung, bronchus and vessels with malposition of the heart in the right hemithorax. (Seven months’ boy)

According to Boyden classification; two patients had type I pulmonary agenesis (agenesia) which was confirmed by total absent of unilateral lung, main bronchus and its pulmonary vessels. Two boys of four months and seven months of age with respiratory distress and history of recurrent chest infection showed evidence of right lung agenesis with mediastinal shift and right side position of the heart in both patients (Fig. 1, 1b and Fig. 2).

**Fig. 3:**
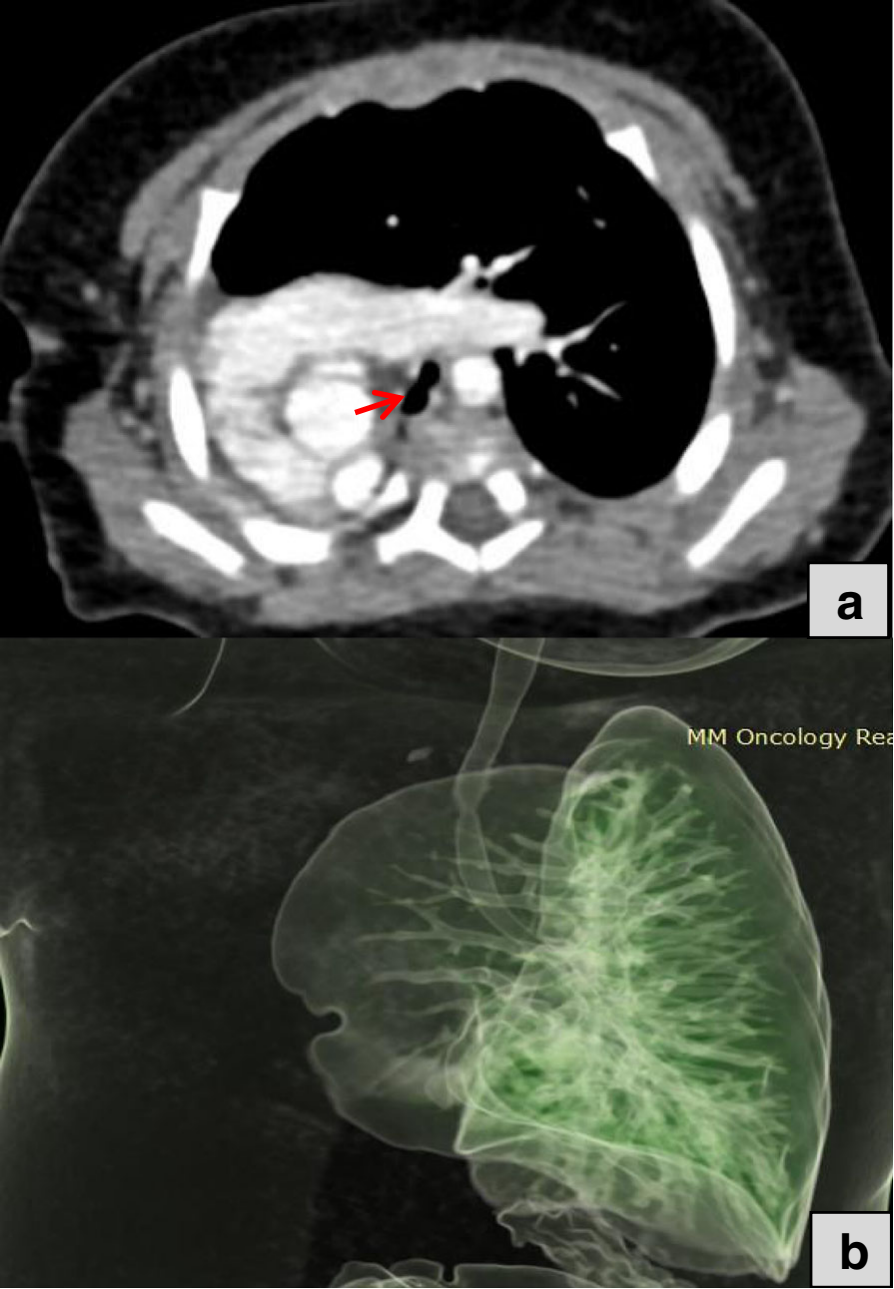
**a** Axial CT image of three months old baby in mediastinal window shows absent right lung with rudimentary right main bronchus (red arrow) associated with right cardiac and great vessels position with extension of left lung towards the right side. Fig. 3:**b** Volume rendered image shows unilateral left lung with complete absent right lung and its bronchus in seven months baby

**Fig. 4:**
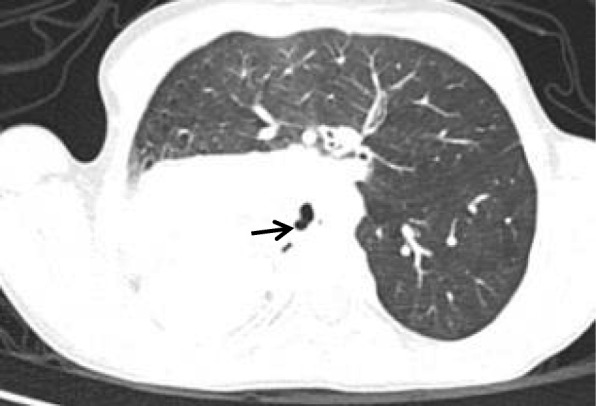
Axial lung window CT images also demonstrating rudimentary right bronchus (black arrow) with absent right lung and herniation of left lung to the right side

**Fig. 5:**
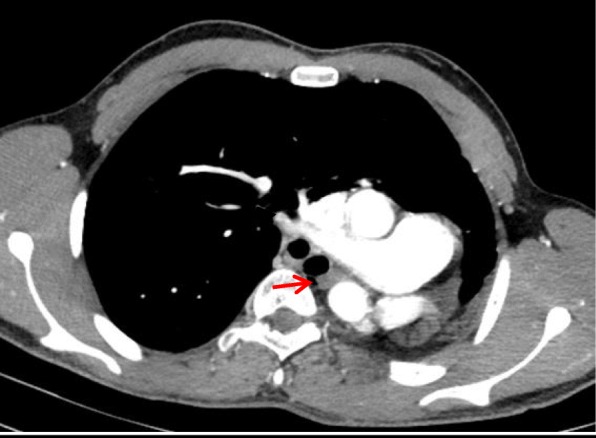
Axial CT image of eighteen years old boy in mediastinal window shows absent left lung with rudimentary left bronchus (red arrow) with left posterior position of cardiac and great vessels and right lung significant extension towards the left hemithorax

Three patients had type II pulmonary agenesis (aplasia) which is characterized by complete absence of unilateral lung and its pulmonary vessels with a small rudimentary blind ended main bronchus. A boy of three months of age with respiratory distress demonstrated right side aplasia (Fig. 3 and Fig.3b). Another case of right lung aplasia with mediastinal shift was noted in a seven years old girl whom presented with dyspnea and chest infection (Fig. 4). Third patient was a young boy of eighteen years old with left lung aplasia and left sided heart with mild kyphoscoliotic changes along the thoracic spine (Fig. 5).

**Fig. 6:**
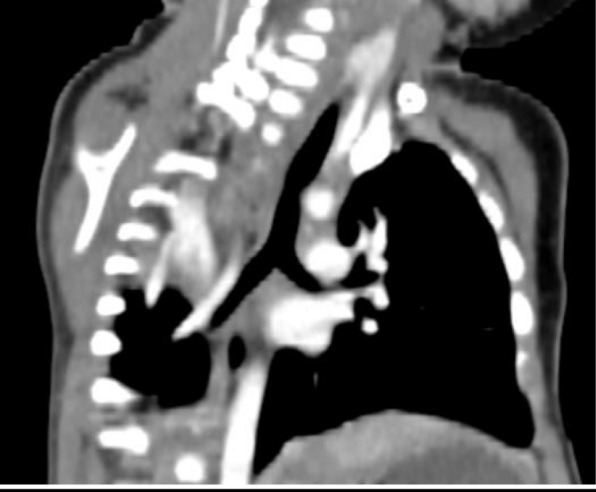
Coronal CT image of one year old boy in mediastinal window shows partial absent right lung (upper and middle) with existence of right lower lobe with its bronchus. The heart locates in the right upper zone

Type III pulmonary agenesis (hypoplasia) was observed in a one year old boy with mild shortness of breath and opacity in the right upper lung zone. The chest CT images demonstrated partial right lung in the lower zone with displacement of heart in the right upper lung (Fig. 6).

## Discussion and conclusion

For the first time pulmonary agenesis was classified by Schneider [8] which later on was modified by Boyden [9] into three groups according to development of their primitive lung bud. Type I which is called pulmonary agenesis is complete absence of unilateral lung parenchyma, its bronchus and vasculature. Type II is named pulmonary aplasia which is complete absence of unilateral lung with a rudimentary bronchus. Type III is pulmonary hypoplasia characterized by partial existence of branchial tree with some parts of unilateral pulmonary parenchyma and its vessels [2]. Although the main etiology of the disease is unknown, lack of vitamin A during pregnancy, viral agents, genetic as well as iatrogenic factors [10] have been mentioned as possible causes [2]. The lungs normally develop from foregut during the 4th and 5th weeks of gestation. The failure of bronchial analogue to divide equally between two lungs with possible abnormal blood flow in dorsal aortic arch during this period may result in hypoplasia, aplasia and agenesis of unilateral pulmonary parenchyma. In the meantime the contra lateral lung produces almost twice alveoli in compensation [11]. As during this period of time the migration of heart also occurs, therefore some cases may coexist with congenital heart anomalies [10]. Pulmonary hypoplasia may occur due to secondary reasons as well such as chest wall deformity, diaphragmatic hernia, cystic adenomatoid malformations, and pleural effusion. Bilateral pulmonary hypoplasia can also happen due to thoracic dystrophies and oligohydramnios [6]. For diagnosis of pulmonary agenesis different imaging techniques can be used. Plain chest shows unilateral opaque lung with mediastinal shift whereas for final diagnosis CT scan, MRI [12], bronchography, bronchoscopy and pulmonary angiography are used. Sometimes the disease can be detected in prenatal life by help of prenatal ultrasound showing hyperechoic hemithorax however the definitive diagnosis is hard [13] which can be confirmed by Fetal MRI [12]. According to the literature, left side agenesis is more common comparing to the right side with longer life expectancy. However in our cases just one patient had left lung agenesis while the other five cases had right side agenesis. Right lung agenesis happens with more incidences of cardiovascular abnormalities and patient may have more severe symptoms due to pronounced carina malformation and cardiac and mediastinal shift [10]. Treatment strategies contain medical management and surgical repair. Medical treatments comprise control of recurrent chest infection, bronchodilators and controlling other complications. Surgery is usually needed in associated congenital anomalies. The prognosis usually depends to functionality of the unilateral existed lung and associated anomalies [2].

As this anomaly can occur at any age, the possibility of lung agenesis should be in differential diagnosis of patients having decrease to absent breath sounds with less or no movement of unilateral chest wall and opaque hemithorax in plain film. For confirmation, diagnostic imaging such as chest CT scan, MRI, bronchoscopy and chest angiography can be done. The early detection of the pulmonary agenesis is essential to reduce the development of fibrosis in patient’s unilateral lung which can occur as result of recurrent chest infection. The surgical procedures should also be in consideration in presence of other congenital anomalies or complications.

## Abbreviations

CT: Computed tomography
MRI: Magnetic resonance imaging

## References

[1] Biyyam Deepa R., Chapman Teresa, Ferguson Mark R., Deutsch Gail, Dighe Manjiri K. (2010). Congenital Lung Abnormalities: Embryologic Features, Prenatal Diagnosis, and Postnatal Radiologic-Pathologic Correlation. RadioGraphics.

[2] Kisku KH, Panigrahi MK, Sudhakar R (2008). Agenesis of lung-a report of two cases. Lung India: official organ of Indian chest. Society.

[3] Johnson RJ, Haworth SG (1982). Pulmonary vascular and alveolar development in tetralogy of Fallot: a recommendation for early correction. Thorax.

[4] Cooney TP, Thurlbeck WM, Mathers J (1985). Lung growth and development in anencephaly and hydranencephaly. Am Rev Respir Dis.

[5] Cooney TP, Thurlbeck WM (1982). Pulmonary hypoplasia in Down's syndrome. N Engl J Med.

[6] Pathania M, Lali BS, Rathaur VK (2013). Unilateral pulmonary hypoplasia: a rare clinical presentation. BMJ case reports.

[7] Katsenos S, Antonogiannaki EM, Tsintiris K (2014). Unilateral primary lung hypoplasia diagnosed in adulthood. Respir Care.

[8] Schneider P, Schawatbe E. E. Die Morphologie der Missbildungen Des Menschen Under Thiere. Jena: Gustav Fischar. 1912;3 Part.2:817–822.

[9] Boyden E. Developmental anomalies. Am J Surg, 1955;89:79–88. [PubMed]. 10.1016/0002-9610(55)90510-9 13218221

[10] Agarwal A, Maria A, Yadav D, Bagri N (2014). Pulmonary agenesis with Dextrocardia and hypertrophic cardiomyopathy: first case report. J Neonatal Biol.

[11] Yetim TD, Bayaroğullari H, Yalçin HP, Arιca V, Arιca SG. Congenital agenesis of the left lung: a rare case. J clin. imag scie. 2011:1. 10.4103/2156-7514.85175 PMC3205521 22059149

[12] Kuwashima S, Kaji Y (2010). Fetal MR imaging diagnosis of pulmonary agenesis. Magn Reson Med Sci.

[13] Dembinski J, Kroll M, Lewin M, Winkler P (2009). Unilaterale pulmonale Agenesie. Aplasie und Dysplasie Zeitschrift für Geburtshilfe und Neonatologie.

